# Unveiling the Predictive Model for Macrovascular Complications in Type 2 Diabetes Mellitus: microRNAs Expression, Lipid Profile, and Oxidative Stress Markers

**DOI:** 10.3390/ijms252111763

**Published:** 2024-11-01

**Authors:** Ayauly Duisenbek, María D. Avilés Pérez, Miguel Pérez, José Miguel Aguilar Benitez, Víctor Roger Pereira Pérez, Juan Gorts Ortega, Botagoz Ussipbek, Arailym Yessenbekova, Gabriela C. López-Armas, Nurzhanyat Ablaikhanova, Fabiola Olivieri, Germaine Escames, Darío Acuña-Castroviejo, Iryna Rusanova

**Affiliations:** 1 Department of Biophysics, Biomedicine and Neuroscience, Al-Farabi Kazakh National University, Al-Farabi Av. 71, Almaty 050040, Kazakhstan; aduisenbek@correo.ugr.es (A.D.); ussipbek.botagoz@kaznu.kz (B.U.); arailym.yesenbekova@kaznu.kz (A.Y.); nurzhanat.ablaihanova@kaznu.kz (N.A.); 2 Endocrinology and Nutrition Unit, Instituto de Investigación Biosanitaria de Granada Ibs.GRANADA, University Hospital San Cecilio, 18007 Granada, Spain; mariolaviles@live.com; 3 Hospital Alto Guadalquivir, 23740 Andujar, Spain; miguel.perez.porras.sspa@juntadeandalucia.es; 4 Hospital de Alta Resolución de Alcalá la Real, 23680 Alcalá de Real, Spain; josemiguel.aguilar.sspa@juntadeandalucia.es; 5 Department of Biochemistry and Molecular Biology I, Faculty of Science, University of Granada, 18071 Granada, Spain; vrpereiraperez@correo.ugr.es (V.R.P.P.); juangorts@correo.ugr.es (J.G.O.); 6 Departamento de Investigación y Extensión, Centro de Enseñanza Técnica Industrial, C. Nueva Escocia 1885, Guadalajara C.P. 44638, Mexico; glopez@ceti.mx; 7 Department of Clinical and Molecular Sciences, Disclimo, Università Politecnica delle Marche, 60126 Ancona, Italy; f.olivieri@staff.univpm.it; 8 Advanced Technology Center for Aging Research, IRCCS INRCA, 60121 Ancona, Italy; 9 Centro de Investigación Biomédica en Red Fragilidad y Envejecimiento Saludable (CIBERFES), Instituto de Salud Carlos III, 28029 Madrid, Spain; gescames@ugr.es (G.E.); dacuna@ugr.es (D.A.-C.); 10 Instituto de Investigación Biosanitaria ibs. GRANADA, Hospital Universitario San Cecilio, 18016 Granada, Spain; 11 Centro de Investigación Biomédica, Instituto de Biotecnología, Parque Tecnológico de Ciencias de la Salud, Universidad de Granada, 18016 Granada, Spain; 12 Department of Physiology, Faculty of Medicine, University of Granada, 18016 Granada, Spain

**Keywords:** type 2 diabetes mellitus, cardiovascular disease, T2DM macrovascular complications, microRNA, oxidative stress, inflammation

## Abstract

To assay new circulating markers related to macrovascular complications (MVC) in type 2 diabetes mellitus (T2DM), we carried out a descriptive cross-sectional study. We recruited 30 controls (CG), 34 patients with T2DM (DG), and 28 patients with T2DM and vascular complications (DG+C); among them, 22 presented MVC. Peripheral blood was used to determine redox status (superoxide dismutase, SOD; catalase, CAT; glutathione reductase, GRd; glutathione peroxidase, GPx; glucose-6-phosphate dehydrogenase, G6PD) and markers of oxidative damage (advanced oxidation protein products, AOPP; lipid peroxidation, LPO), nitrite levels in plasma (NOx). Inflammatory markers (IL-1β, IL-6, IL-10, IL-18, MCP-1, TNF-α) and the relative expression of c-miRNAs were analyzed. The real-time PCR results showed that the expressions of miR-155-5p, miR-21-5p, miR-146a-3p, and miR-210-3p were significantly higher in the DG group compared to the CG. The DG+C group presented statistically relevant differences with CG for four miRs: the increased expression of miR-484-5p, miR-21-5p, and miR-210-3p, and decreased expression of miR-126a-3p. Moreover, miR-126a-3p was significantly less expressed in DG+C compared to DG. The application of binary logistic regression analysis and construction of receiving operator characteristic curves (ROC) revealed two models with high predictive values for vascular complications presence: (1) HbAc1, creatinine, total cholesterol (TC), LPO, GPx, SOD, miR-126, miR-484 (Exp(B) = 0.926, chi^2^ = 34.093, *p* < 0.001; AUC = 0.913). (2) HbAc1, creatinine, TC, IL-6, LPO, miR-126, miR-484 (Exp(B) = 0.958, Chi^2^ = 33.863, *p* < 0.001; AUC = 0.938). Moreover, our data demonstrated that gender, TC, GPx, CAT, and miR-484 were associated with MVC and exhibited higher predictive values (Exp(B) = 0.528, *p* = 0.024, Chi^2^ = 28.214, AUC = 0.904) than classical variables (Exp(B) 0.462, *p* = 0.007, Chi^2^ = 18.814, AUC = 0.850). miR-126, miR-484, IL-6, SOD, CAT, and GPx participate in vascular damage development in the studied diabetic population and should be considered for future studies.

## 1. Introduction

Cardiovascular disease (CVD) is a major vascular complication related to type 2 diabetes mellitus (T2DM) and is connected to the high mortality rate of these patients [1]. To date, there are no reliable markers that allow identifying patients at risk of developing CVD in diabetes, nor are there an effective treatment aimed at reducing their risk [2]. Vascular complications include microangiopathies (retinopathy, neuropathy, and nephropathy) and macroangiopathies, including ischemic heart disease, peripheral arterial vascular disease, and stroke—all of which are considered to be the result of endothelial dysfunction (ED) [3]. Clinical studies show that strict glycemic control slightly reduces diabetic vascular complications [4]. Vascular damage progresses when the patient remains asymptomatic; if the diagnosis is not realized in time, the cells acquire a metabolic memory, changing their metabolism, which is difficult to correct later [5]. The mechanism of ED onset involves several processes, including high oxidative stress due to metabolic changes experienced by endothelial cells under hyperglycemia combined with chronic inflammation. Communication between cells also changes, and several studies have tried to detect different circulating and exosomal microRNAs (miRNAs) related to the propagation of inflammation and the development of vascular damage [6,7].

MicroRNAs, circulating small non-coding RNA molecules, have emerged as critical regulators and potential biomarkers in understanding these complex facets of T2DM [4]. Diabetes interferes with the intricate equilibrium of endothelial function. Likewise, the inflammatory milieu exacerbates endothelial dysfunction, amplifies vascular permeability, and initiates atherogenic processes [8]. miRNAs have been evaluated in various in vitro and in vivo research models related to T2DM [9]. These models often include cell cultures and animal models that simulate the pathophysiological aspects of diabetes. In cases of T2DM, dysregulated miRNAs can modulate insulin signaling pathways, compromising glucose homeostasis [10]. At the same time, abnormal miRNA expression can also affect lipid metabolism, a significant factor in developing dyslipidemia, a characteristic of cardiovascular disease [11]. Above all, its participation is notable in regulating inflammatory and oxidative processes in cells and their release into the circulation. Several of these miRNAs were studied, but more studies are necessary to correlate the miRNAs expression, oxidative and inflammatory status, with macrovascular complications. In this study, we included miR-21, linked to chronic inflammation, and participated in the suppression of antioxidant signaling. MiR-146a plays a role in the modulation of the NF-κB signaling pathway, exercising its anti-inflammatory function. miR-155 can target multiple antioxidant enzymes and play a significant role in maintaining adipose tissue metabolism. A pro-oxidant effect through the regulation of Nrf2 is exerted by miR-27a, which can contribute to vascular damage development. MiR-210 has multiple effects on different tissues: it can activate endothelial progenitor cells to differentiate into endothelial cells, it is considered hypoxa-miRNA in muscles, and it also induces macrophage inflammatory polarization, contributing to chronic inflammation. MiR-126 has an antioxidative protective role on endothelial cells, and miR-484 in diabetes is known since it may be a regulator of insulin expression by decreasing it in the β pancreatic cells in response to increased glucose. Also, miR-484 is an interesting miRNA for study because it regulates mitochondria function and acts on the endothelial nitric oxide synthase (eNOS) in hyperglycemic conditions.

Starting from the previous knowledge that circulating miRNAs, oxidative stress, and inflammatory events are involved in the pathogenesis of vascular damage leading to vascular complications, including macrovascular complications, we hypothesize that the analysis of selected miRNAs (miR-21, miR-126, miR-146a, miR-155, miR-27a, miR-210, and miR-484), together with oxidative stress markers, inflammatory parameters, and demographic/clinical variables in individuals involved in this study, may help to identify risk factors related to vascular complications, including macrovascular events in diabetic studied population. For this, we used a descriptive cross-sectional design, recruiting three groups of participants. If identified, these markers may serve as potential biomarkers for T2DM-related complications and offer new avenues for diagnostic and therapeutic intervention.

## 2. Results

### 2.1. Characteristics of the Studied Population

The profiles and biochemical parameters of the participants are presented in Table 1. We recruited 18 women and 12 men in the control group, and 18 women and 16 men in the patients with diabetes and without vascular complications (DG group). These two groups were matched by gender (chi^2^ = 0.323, *p* = 0.570). Nevertheless, the group of diabetics with complications (DG+C) had a higher number of male participants (5 women versus 23 men), so the gender variable influenced the model of vascular complications. It is worth noting that all groups were recruited in the endocrinology clinic for 10 months. The control group recruitment was controlled because volunteers from different institutions participated, always maintaining the parity criteria: gender, age, and BMI. The patient groups were recruited according to the influx of patients in the indicated period during the consultations of the physicians who participated in the study.

In the control group, most participants (22 participants) were in the age range of 40–55 years, compared with 6 participants in the 56–70 range, and 2 participants who were older than 71 years. It is to be expected that the diabetic patients were older: 37 participants were in the range of 56–70 years, while the 40–55 range included 9 participants, and 16 participants were over 71 years. However, among people with diabetes, both with and without complications, there was no significant difference in the age ranges (range 56–70 years: 21 in DG and 16 in DG+C; range 40–55 years: 5 in DG and 4 in DG+C; range over 71 years: 8 in DG and 8 in DG+C), or in mean age (63.47 ± 1.30 in DG and 64.96 ± 1.68 in DG+C). Therefore, age was not a significant variable in evaluating the predictive model of vascular complications.

Regarding BMI, the control group included 16 individuals who were a normal weight, 11 who were overweight, and 3 with obesity. As expected, people with diabetes had higher body mass indexes: 14 were overweight in the DG group and 16 in the DG+C group. Obese individuals were 13 in the DG group and 11 in the DG+C group. However, it is worth noting that among all patients with diabetes, BMI was matched between the two groups (DG and DG+C): chi^2^ = 4.259, *p* = 0.119.

Physical activity was classified as:

Low intensity: Activities that do not significantly elevate the heart rate, such as slow walking or stretching. Moderate intensity: Activities that increase the heart rate and breathing, such as brisk walking or bicycling. High intensity: Activities that require considerable effort, such as running, or interval training. As expected, significant differences were observed between the three groups (chi^2^ = 25.465, *p* < 0.001). However, physical activity did not differ between the two groups among all patients with diabetes (chi^2^ = 3.791, *p* = 0.188), or in patients who had macro complications: chi^2^ = 2.295, *p* = 0.317.

Both diabetic groups had higher mean HbA1c and glucose levels than the control group and an increased HOMA-IR index, indicating greater insulin resistance. Regarding lipid profile, total cholesterol and LDL levels were lower in the diabetic groups, while triglyceride (TG) levels were higher in both compared to the CG. Additionally, HDL levels were reduced in both diabetic groups. These findings suggest that participants with diabetes have poorer glycemic control and increased insulin resistance but exhibit changes in lipid profiles despite having increased body mass index (BMI).

Table 2 outlines the drug therapies used for patients with microangiopathies and CVD. Both groups used several glucose-lowering medications, including metformin and a range of prevalent antihyperglycemic agents. Insulin therapy was more common in the CVD group. Cholesterol-lowering treatment was more frequently administered in the microangiopathy group, whereas anticonvulsants were used in the CVD group. Our analysis of the contingency tables has not revealed a statistical relationship between the treatments and vascular complications.

### 2.2. Differential Patterns of miRNA Expression in Studied Population

**Figure 1:**
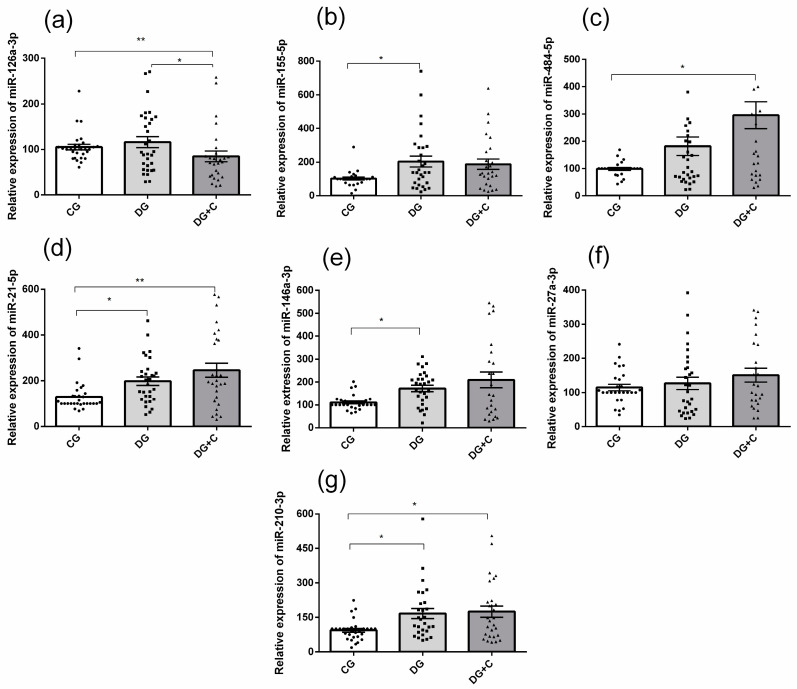
The relative expression of (**a**) miR-126, (**b**) miR-155, (**c**) miR-484, (**d**) miR-21, (**e**) miR-146a, (**f**) miR-27a, and (**g**) miR-210 in plasma of controls (CG), T2DM patients without complications (DG), diabetics with complications (DG+C). Data are presented as means ± standard error of the mean (SEM). *p* values from Kruskal–Wallis test. Comparisons between groups are indicated in the graphs. * *p* < 0.05, ** *p* < 0.01.

The real-time PCR results showed that the plasma levels of miR-155-5p, miR-21-5p, miR-146a-3p, and miR-210-3p were significantly higher in the DG group compared to the control group (*p* < 0.05) (Figure 1b,d,e,g). DG+C group presented statistically relevant differences compared to CG for four miRNAs: the expression was increased for miR-484-5p (Figure 1c), miR-21-5p (Figure 1d), and miR-210-3p (Figure 1g), but it was decreased for miR-126a-3p (Figure 1a). Moreover, miR-126a-3p was significantly less expressed in DG+C compared to DG (Figure 1a). Only miR-27a-3p did not present significant differences in its relative expression.

### 2.3. Oxidative Status Markers in Studied Samples

**Figure 2:**
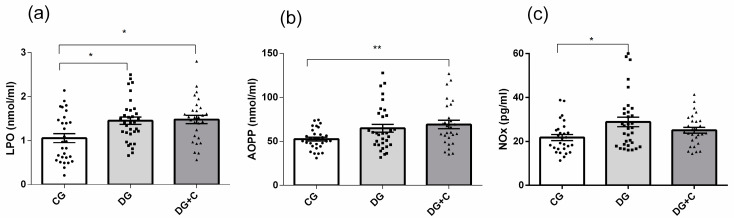
Assessment of oxidative stress markers in plasma from diabetic patients without complications (DG), with vascular complications (DG+C), and controls (CG): (**a**) Advanced oxidation protein products (AOPP), (**b**) lipid peroxidation (LPO), and (**c**) nitric oxide (NOx). Data are presented as means ± standard error of the mean (SEM). *p* values from Kruskal–Wallis test. Comparisons between groups are indicated in the graphs. * *p* < 0.05 and ** *p* < 0.01.

Determination of the extracellular oxidative status, which reflects the damage produced in the blood cells’ balance between oxidant production and antioxidant defenses, was conducted by analyzing plasma LPO (lipid peroxidation), AOPP (advanced oxidation protein products), and NOx (nitrite) levels. DG and DG+C patients showed substantially increased levels of all two markers, with *p* < 0.05 for LPO and *p* < 0.01 for AOPP, compared to the CG (Figure 2a,b). In DG patients, NOx and LPO levels were increased (Figure 2a,c).

**Figure 3:**
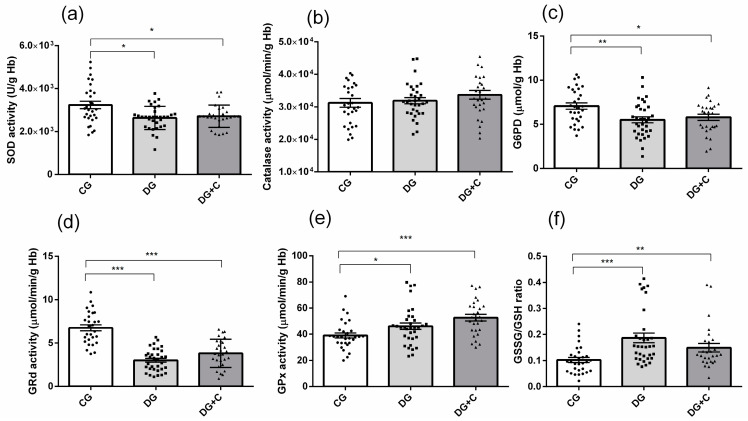
Assessment of oxidative stress markers in erythrocytes from diabetic patients without complications (DG), with vascular complications (DG+C), and controls (CG): (**a**) superoxide dismutase (SOD), (**b**) catalase (Cat), (**c**) glucose-6-phosphate dehydrogenase (G6PD), (**d**) glutathione reductase (GRd), (**e**) glutathione peroxidase (GPx), and (**f**) glutathione disulfide to reduced glutathione ratio (GSSH/GSH). Data are presented as means ± standard error of the mean (SEM). *p* values from Kruskal–Wallis test. Comparisons between groups are indicated in the graphs. * *p* < 0.05, ** *p* < 0.01, *** *p* < 0.001.

Determination of intracellular oxidative status included the measurement of antioxidant enzyme activities (SOD, CAT, and G6PD) and glutathione cycle components (GSSG, GSH, GRd, and GPx) in erythrocytes. In terms of antioxidant enzymes, there was a significant decrease in SOD activity in both diabetic groups (*p* < 0.05) (Figure 3a). Catalase activity showed no significant differences between the groups (Figure 3b), while G6PD activity was lower in the DG and DG+C groups compared to the control group (*p* < 0.01, *p* < 0.05, respectively) (Figure 3c).

GRd activity was markedly decreased in both diabetic groups compared to controls (*p* < 0.001, Figure 3d), whereas GPx activity was significantly increased in the same groups (*p* < 0.001 and *p* < 0.05, respectively) (Figure 3e). Finally, the GSSG/GSH ratio was markedly increased in the DG group (*p* < 0.001) and in the DG+C group (*p* < 0.01) in comparison to the control group (Figure 3f).

### 2.4. Inflammatory Status and Correlations Between Studied Markers and Clinical Profile

**Figure 4:**
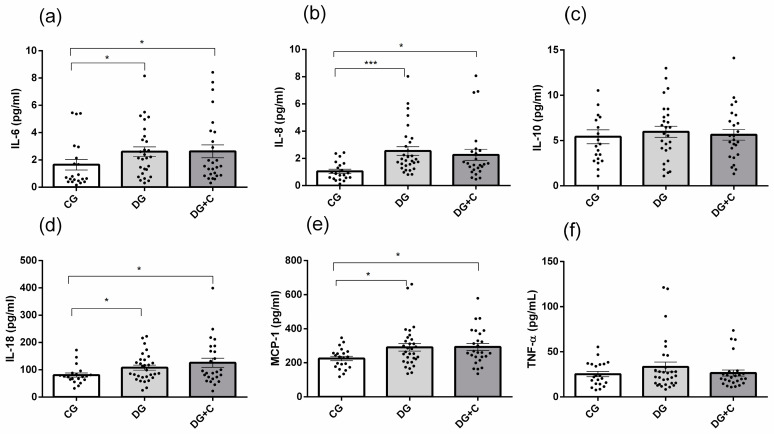
The concentration of pro- (IL-6, IL-8, IL-18, MCP-1, and TNF-α) and anti-inflammatory cytokines (IL-10) in plasma samples from diabetic and control groups. (**a**) IL-6; (**b**) IL-8; (**c**) IL-10; (**d**) IL-18; (**e**) MCP-1 and (**f**) TNF-α were assessed. Data are presented as means ± standard error of the mean (SEM). *p* values from Kruskal–Wallis test. Comparisons between groups are indicated in the graphs. * *p* < 0.05 and *** *p* < 0.001.

Among inflammatory markers measured in three studied groups, significantly increased levels were detected for IL-6, IL-8, IL-18, and MCP-1 in the DG and DG+C groups compared to the CG group (Figure 4a,b,d,e). However, no significant difference was observed between the DG and DG+C groups. IL-10 and TNF-α did not show significant differences among the CG, DG, and DG+C groups.

Pearson correlations analysis revealed the following significance between oxidative stress status, inflammatory markers, and biochemical variables in diabetic patients:

Positive correlations between:

LPO and total cholesterol (r = 0.278, *p* = 0.032).

LPO and TG (r = 0.285, *p* = 0.029).

LPO and HOMA-IR (r = 0.281, *p* = 0.036).

AOPP and TG (r = 0.763, *p* < 0.001).

GRd and HOMA-IR (r = 0.255, *p* = 0.050).

And negative correlations between:

CAT and total cholesterol (r = −0.363, *p* = 0.004)

SOD and total cholesterol (r = −0.272, *p* = 0.035)

IL-10 and HOMA-IR (r = −0.300, *p* = 0.036)

TNFα and HOMA-IR (r = −0.248, *p* = 0.050)

Then, the dates of diabetic patients were adjusted for gender, age, and BMI. The negative correlations between CAT and TC, CAT and LDL, IL-10, and HOMA-IR were conserved. Moreover, other correlations were found (see Table 3).

Here, we evaluated possible correlations between the miRNAs expression profile, biochemical parameters, and inflammatory markers. Firstly, we correlated these parameters in all participants and detected the following correlations:

MiR-21 showed a significant positive correlation with Glucose (r = 0.295, *p* = 0.006), HbAc1 (r = 0.272, *p* = 0.012), and a negative correlation with total cholesterol (r = −0.231, *p* = 0.033) and LDL (r = −0.275, *p* = 0.011).

MiR-126 presented a positive correlation with TG (r = 0.344, *p* = 0.001).

Mir-146a presented positive correlations with glucose (r = 0.491, *p* < 0.001), and HbAc1 (r = 0.326, *p* = 0.003).

MiR-155-5p showed a significant positive correlation with HbAc1 (r = 0.251, *p* = 0.021), and with glucose (r = 0.471, *p* < 0.001).

MiR-484 was negatively correlated with total cholesterol (r = −0.287, *p* = 0.009), and with LDL (r = −0.293, *p* = 0.007).

MiR-210 showed a positive correlation with HbAc1 (r = 0.266, *p* = 0.014), but a negative correlation with total cholesterol (r = −0.359, *p* = < 0.001), LDL (correlation = −0.356, *p*-value < 0.001), and TNF-α (correlation = −0.239, *p*-value = 0.042).

**Figure 5:**
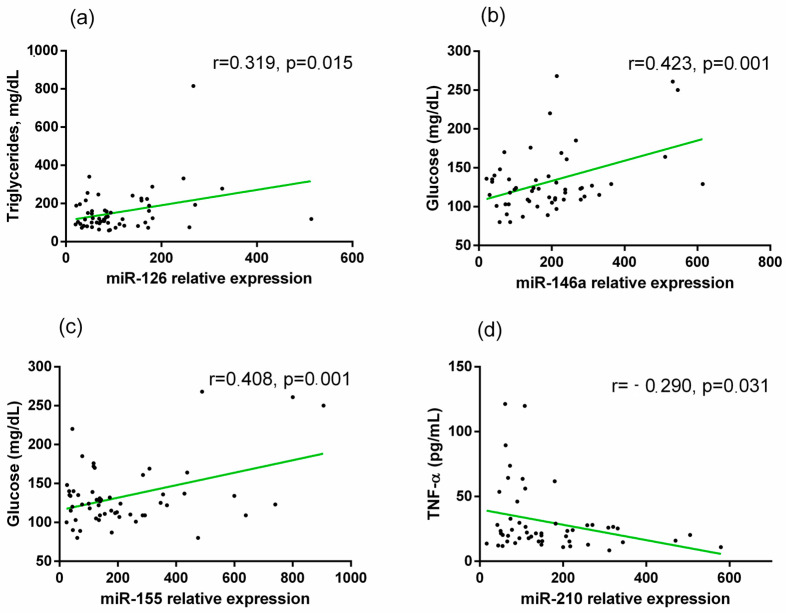
Correlations between relative (**a**) miR-126 and TG, (**b**) miR-146a and glucose, (**c**) miR-155 and glucose, and (**d**) miR-210 and TNF-α in diabetic patients. Pearson correlation coefficient (r) analysis was used.

In diabetic patients, we detected a positive correlation between miR-126 and TG (r = 0.413, *p* = 0.001), between miR-146a and glucose levels (r = 0.424, *p* = 0.002), and miR-155 was positively correlated with glucose (r = 0.408, *p* = 0.002). Otherwise, miR-210 showed a negative correlation with TNF-α (r = −0.290, *p* = 0.037) (Figure 5a–d).

After we adjusted all diabetic patients’ parameters for gender, BMI, and age, two correlations were observed: between miR-146a and glucose (r = 0.376, *p* = 0.007) and between miR-155 and glucose (r = 0.409, *p* = 0.004).

Also, we analyzed the correlations between the expressions of miRNAs and markers of oxidative and inflammatory status in diabetic patients, and it was adjusted for age, gender, and BMI (Table 4). It should be noted that in both analyses, there is a positive correlation between miR-21 and LPO, between miR-126 and LPO, between miR-27a and LPO, and between miR-210 and CAT. In addition, a negative correlation between miR-21 and SOD was found.

### 2.5. Evaluation of the Diagnostic Accuracy of Biomarkers Related to Diabetes and Macrovascular Complications in T2DM

**Figure 6:**
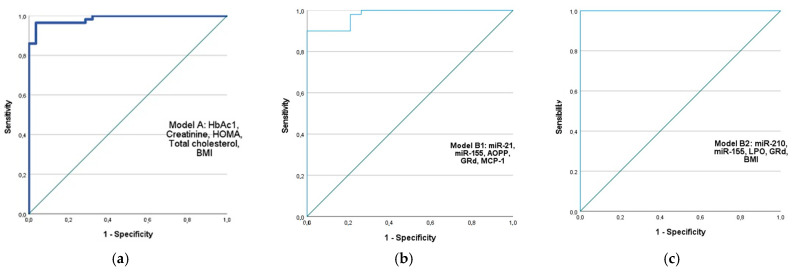
Receiver operator characteristic (ROC) curves generated for sensitivity analysis show diagnostic performances of plasma markers for diabetes. AUC of proposed models: (**a**) AUC (95%) = 0.986; creatinine, HOMA-IR, HbA1c, cholesterol and BMI; (**b**) AUC (95%) = 0.978; miR-21, miR-155, AOPP, GRd, MCP-1; (**c**) AUC (95%) = 1.000, include miR-155, miR-210, LPO, GRd and BMI. AUC- area under the curve.

We performed the binary logistic regression analysis to find markers with significant predictive value for diabetes development (*p*-value and Exp(B)). Receiving operator characteristic (ROC) curves were generated to facilitate a more visual and direct comparison between three selected models (Figure 6):

Model A includes classical biochemical parameters for diabetes diagnosis: HbA1c, creatinine, TC, HOMA-IR, and BMI were selected. The following values were obtained for this model: AUC (95%) = 0.986, Exp(B) = 2.036, Chi^2^ = 71.008, *p* < 0.001, Cox and Snell R^2^ = 0.566, and Nagelkerke R^2^ = 0.788.

We proposed two models B that included the following:

Model B1: miR-21, miR-155, AOPP, GRd, MCP-1: AUC (95%) = 0.978, Exp(B) = 2.632, Chi^2^ = 57.645, *p* < 0.001, Cox and Snell R^2^ = 0.566, and Nagelkerke R^2^ = 0.819.

Model B2: miR-210, miR-155, LPO, GRd, BMI: AUC (95%) = 1.000, Exp(B) = 2.037, Chi^2^ = 103.920, *p* < 0.001, Cox and Snell R^2^ = 0.718, and Nagelkerke R^2^ = 1.000.

We found that the combination of miR-210 and miR-155 expression levels with LPO, GRd, and BMI had a predictive value for the development of diabetes that is similar to, or greater than, that of a classical diagnostic parameter, including HbAc1. Moreover, future studies could consider miR-21, AOPP, and MCP-1 independent predictors of T2DM risk.

To identify markers related to vascular complications in diabetic patients, we performed binary logistic regression analysis for each marker. We then selected those markers with a significant *p*-value and AUC values greater than 0.650. We constructed three models, one of which included classical parameters (Model 1): Glucose, HbAc1, creatinine, HOMA-IR, TC, TG, and gender. Another two models included HbAc1, creatinine, TC, and newly studied markers (Models 2 and 3). In Table 5, we described the statistical dates of these three models:

Finally, to identify markers related to macrovascular complications (MVC), we performed binary logistic regression analysis on all diabetic patients (62 patients: 34 patients with T2DM without complications (DG), plus 28 patients with T2DM and vascular complications (DG+C), relating them to those who had macrovascular complications at the time of the study. We then conducted a Receiver Operating Characteristic (ROC) curve analysis, In Table 6, the Area Under the Curve (AUC), Odds Ratio (OR), and *p*-values were included. In Table 6, the predictive values for the markers that meet the statistical parameters are marked in bold. We selected those markers with a significant *p*-value and AUC values greater than 0.600. Based on these results, we constructed the ROC curve for the model that included TC, GPx, CAT, Gender, and miR-484.

**Figure 7:**
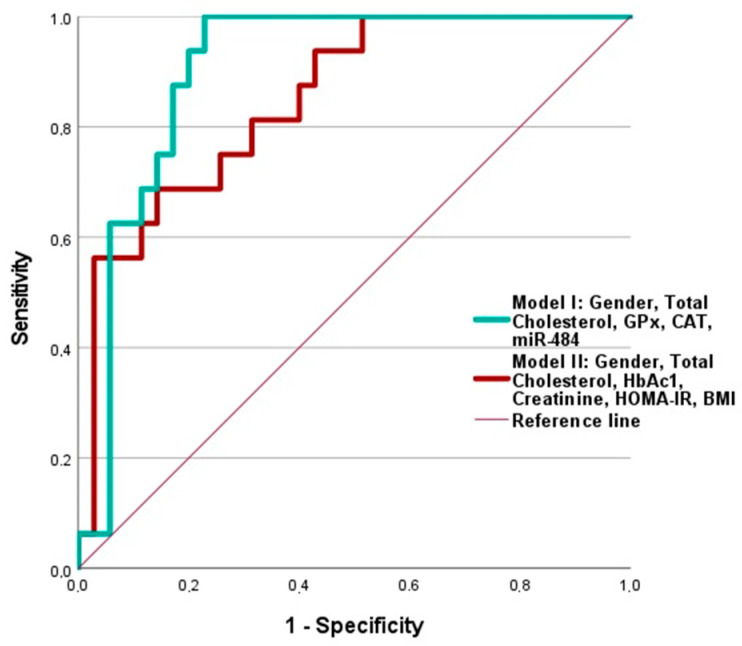
Receiver-operating characteristic (ROC) curve analysis of two models proposed for significantly predicting the risk of development macrovascular complications in the studied diabetic patients. Model I included a combination of assayed predictors: gender, total cholesterol, GPx, CAT, miR-484 (Exp(B) 0.528, *p* = 0.024, Chi^2^ = 28.214, AUC = 0.904(95%). Model II included classical variables: gender, total cholesterol, HbAc1, creatinine, HOMA-IR, BMI (Exp(B) 0.462, *p* = 0.007, Chi^2^ = 18.814, AUC = 0.850(95%).

We found that the combination of detected markers was able to significantly predict the risk of developing macrovascular complications in diabetics, even with a higher predictive value than the model that included the classic parameters: HbAc1, creatinine, HOMA-IR, BMI, sex, and total cholesterol. The application of ROC curve analysis to this model to evaluate its predictive value resulted in an area under the curve (AUC) of 0.904, while the AUC for the classic parameters was 0.850 (Figure 7).

## 3. Discussion

In this study, we counted 22 patients with macrovascular complications among 62 patients with diabetic disease. We analyzed the circulating markers’ relationship with all cardiovascular complications, including macrovascular complications.

A comprehensive analysis of the redox status, antioxidant system, and oxidative damage in blood samples revealed significant increases in LPO and AOPP levels in patients with T2DM with complications compared to the control group, indicating the deterioration of vascular status caused by the disease, which corresponds to studies in animal models [12] and in diabetic patients [13]. In the present study, we found an increase in nitrite and nitrate (NOx) in DG, but this increase was not observed in DG with VCs. The NOx are indirect products of nitric oxide synthase (NOS), and we cannot confirm that these molecules are the results of inducible NOS (iNOS). Still, we can ensure that iNOS isoenzymes are induced in proinflammatory conditions, which is expected in diabetes [14].

The antioxidant defense of diabetic patients was measured by activities of SOD, CAT, and GRd. The SOD enzyme is the first antioxidant defense, and its activity was decreased in both groups of patients. This result is consistent with our previous study in the Mexican population [15]. Thus, we can infer that both the European and Mexican populations share specific characteristics of T2DM in addition to those already well described, which may well be related to short-chain fatty acids (SCFA) and branched-chain amino acids (BCAA) that modify the intestinal microbiota causing dysbiosis and increasing oxidative stress in T2DM [16]. In this study, we did not analyze the participants’ diets, but emerging studies have shown that an altered microbiota composition related to a high-fat diet and SCFA can alter intestinal permeability and trigger an immune response [2]. Cu/Zn dependent cytoplasmatic SOD is an enzyme sensitive to high oxidative stress. And although there are studies that have detected an increase in SOD [17], most of the studies in human samples and animal diabetic models also reported decreased SOD activity [18]. Although our previous study [15] reported an increased CAT activity, we did not find differences between the three groups in Spanish participants and diabetic patients. However, within the group of diabetics, patients who had macro complications presented CAT values significantly higher than the rest of the patients: without complications vs. with macro complications: 34939.7 ± 1399.6, n = 22, vs. 31502.7 ± 894.3, n = 40, *p* = 0.035 (data not presented in results). Also, we detected a negative correlation between CAT and total cholesterol in diabetic patients adjusted for gender and BMI (r = −0.363, *p* = 0.004), which suggests that its lower antioxidant activity is related to the worst lipid profile.

Although catalase is involved in the catalysis of H_2_O_2_ into H_2_O, other enzymes, like GPx and GRd, play a predominant role in the redox state maintenance. However, the binary logistic regression analysis for developing complications in our population calculates that CAT and GPx have a significant predictive value and demonstrate a high AUC value. We observed a decrease in GSH levels in line with the reduction in GRd activity in both diabetic groups, which may be partly related to the reduction in NADPH availability. The NADPH may be used by aldose reductase to form sorbitol from glucose in the alternative polyol pathway under hyperglycemic conditions [19]. Furthermore, decreased G6PD activity leads to decreased NADPH levels, limiting GRd activity. Considering the antioxidant metabolism of glutathione, these results would explain the elevated GSSG/GSH ratio in patients with T2DM compared to the control group, an indicator of elevated oxidative stress. In the diabetic group, adjusted for gender and BMI, we found a negative correlation between GPx and cholesterol, and between GPx and HOMA-IR, and a positive correlation between GRd and glucose. Moreover, GPx activity was considered a risk factor in the model for MVCs among diabetic patients.

It has been shown that hyperglycemia induces G6PD activity and expression profiling in different tissues in animal models in the early stages of the disease [20]. However, it seems that the progression of diabetes eventually leads to a decrease in G6PD activity, as we found in this study [15]. Recently, it was demonstrated that individuals of African ancestry with a G6PD deficient risk allele presented an increased risk of diabetes complications, like retinopathy and neuropathy [21]. Although in our study, we did not find a correlation between decreased G6PD activity and glycemic status, the negative correlations between G6PD and total cholesterol and LDL levels point to the relation of this enzyme with metabolic changes. Our study suggests that GPx and GRd should be considered markers requiring further analysis due to their involvement in developing vascular complications.

As expected, this study demonstrates significantly increased IL-6, IL-8, IL-18, and MCP-1 expression in patients with diabetes, with and without VCs, compared to the control group. However, no significant differences existed in IL-1, IL-10, or TNF-α levels. Interestingly, IL-6, a difference from our previous study in the Mexican population, had no significant predictive value for the development of diabetes or cardiovascular complications. This may be because the entire Mexican population had a high BMI; in exchange, the DG+C group had a significantly higher BMI than the control group in the current study, indicating that increasing IL-6 may be related to obesity. The correlations found in diabetic patients between IL-8, IL-18, and molecular damage markers, such as AOPP and LPO, demonstrate their involvement in oxidative damage (Table 3). Notably, MCP-1 showed a negative correlation with GRd and a positive correlation with creatinine levels in diabetics adjusted for gender and BMI (Table 3). In addition, only MCP-1 had a high predictive value for the development of diabetes and CVD, as shown by the regression logistic analysis and the use of this model represented by the ROC curve.

Monocyte Chemoattractant Protein-1 (MCP-1), also known as CCL2, is a chemokine that plays a crucial role in the immune response by recruiting monocytes, memory T cells, and dendritic cells to sites of inflammation [22]. MCP-1 is one of the key mediators of inflammation, and its utility as a biomarker is supported by its ability to reflect the underlying inflammatory state that drives the progression of T2DM and its cardiovascular disease [23]. Moreover, we found a significant negative correlation between MCP-1 levels and GRd activity (MCP-1/GRd: r = −0.339, *p* = 0.025), which points to its participation in the development of oxidative stress. Elevated expression of MCP-1 in adipose tissue and other organs in type 2 diabetes promotes the migration and activation of macrophages, thereby amplifying the inflammatory process through the secretion of pro-inflammatory cytokines and reactive oxygen species (ROS) [24]. We propose to pay attention to the study of this marker. Furthermore, we found that MCP-1 is included in the predictive model for diabetes (Model B1).

We included miR-126 in the proposed model to predict the risk of vascular complications in diabetic patients (Models 2 and 3). For some years now, Olivieri’s studies have shown a significant increase in miR-21-5p and a decrease in miR-126-3p in T2DM with cardiovascular events vs. all the other diabetic patients [25]. Several studies have subsequently been conducted that confirm these findings. According to Dehghani, miR-126 gradually decreases in prediabetic patients and those with T2DM compared to healthy controls [26]. Furthermore, this study suggests a negative correlation between miR-126 expression and NF-kB of peripheral blood mononuclear cells, pointing to its anti-inflammatory effect. miR-126 has a protective role on endothelial cells against oxidative damage, inducing SIRT1 and SOD2 expression, and it is implicated in the positive regulation of the response of endothelial cells to vascular endothelial growth factor (VEGF), which is an angiogenic growth factor involved in mitogenesis and permeability processes [27]. In the current study, c-miR-126 showed decreased expression in DG+C compared to CG and DG. This result is based on several recent studies. In our previous study of the Mexican population, its expression was decreased. Moreover, miR-126 and miR-21, GPx, and AOPP levels were proposed as possible biomarkers of vascular damage in T2DM patients [15]. Some analyses indicate that hyperglycemia reduces the concentration of miR-126 in the heart and plasma, contributing to diabetic microangiopathy and macroangiopathy [28]. MiR-126 presented a positive correlation with TG (correlation = 0.344, *p*-value = 0.001) in all participants and in diabetic patients. This correlation is not maintained in diabetic patients adjusted for gender, age, and BMI, but a new positive correlation between miR-126 and LPO was detected. This study’s specific finding is that the control group has higher cholesterol and LDL levels than the two diabetic groups. This lipid profile is worse than expected in people who do not have diabetes and meet the inclusion criteria, just as patients who have better lipid profile controls have likely undergone cholesterol-lowering therapy. However, oxidative damage in blood samples from patients with diabetes corresponds to the high levels of LPO and the positive correlations between LPO and cholesterol, as well as between LPO and TG.

We demonstrated that circulating levels of miR-155 were up-regulated in plasma derived from diabetic group patients (DG) concerning CG. Likewise, there is a trend of an increase in the DG+C group, but this is not significant. One recent study suggested that c-miR-155-5p can be used as a potential circulating biomarker for T2DM and is especially useful, along with other inflammatory markers, in identifying obese patients with a risk of developing DM2 [29]. Among multiple functions of the miR-155, highly expressed by hematopoietic cells, it is involved in an innate immune response, playing a pro-inflammatory role [30,31]. Nonetheless, miR-155 expression could change depending on the different phases of inflammation [32]. Several studies described the participation of circulating miR-155 in diabetes development: a significant decrease in serum levels of miR-155 has been observed in type 2 diabetes mellitus (DMT2) patients compared to healthy controls [33,34,35]. At the same time, an increase in miR-155 expression has been detected in several studies [36,37], including type 1 diabetes [38]. There is no consensus on the relationship between miR-155 expression levels and diabetic complications, but it seems that miR-155 may play a significant role in maintaining adipose tissue metabolism [39]. Our study detected a positive correlation between miR-155 expression and glucose levels in the entire population, in all diabetics, and the diabetic group adjusted by gender, BMI, and age. Moreover, including miR-155 in the model for diabetic risk assessment significantly increased this model’s significance. This finding correlates with previous data, such as the up-expression of miR-155 in conditions of hyperglycemia found in both in vitro and in vivo studies [40,41]. However, we have not detected that deregulation of miR-155 has significant predictive risk value for macrovascular diabetic complication development.

Previous studies reported the overexpression of miR-210 in the peripheral blood of patients with T2DM [42] and in exosomes from the serum of T2DM patients with obesity [43]. In our study, both groups of diabetics had significantly higher expression of miR-210 than the control group (Figure 1g). Moreover, we found a positive correlation of miR-210 with HbAc1 but a negative correlation with total cholesterol, LDL, and TNF-α. Then, diabetic participants were adjusted for age, gender, and BMI, the correlation between miR-210 and TNF-α was lost. This can point out that glucose and lipid metabolisms are involved in regulating miR-210. Previous studies have found that miR-210 expression was significantly increased in the intimae layer of diabetic patients with atherosclerosis [44] and in the aorta of the animal model of high-fat-fed rats [45]. MiR-210 has a wide range of physiological functions. miR-210 expression was significantly increased, promoting NF-κB dependent proinflammatory cytokine expression and inhibiting SOCS1 (suppressor of cytokine signaling 1), thus inducing macrophage polarization from M2 to M1 state and contributing to a fatty tissue chronic inflammation and insulin resistance that could end up in obesity-induced T2DM [46]. In this study, diabetic patients presented an improved lipid profile, which could have influenced the relationship between miR-210 expression and lipid levels (cholesterol and TG) to be contrary to that expected. Furthermore, the negative correlation between miR-210 and TNF-alpha disappeared when diabetics were adjusted for age, gender, and BMI. Furthermore, it is worth noting that in this last case, miR-210 showed a positive correlation with LPO and CAT, pointing to its possible contribution to the damage caused by diabetes. This statement is reinforced when we see that the B2 model that includes miR-210 and miR-155, LPO GRd, and BMI presents a maximum predictive value for the risk of developing diabetes (AUC = 1.000, Figure 6c).

MiR-146a-3p stands out as one of the initial miRNAs documented as an anti-inflammatory miR with its ability to inhibit NF-κB activation by decreasing the levels of IL-1 receptor-associated kinase 1 (IRAK1) and TNF receptor-associated factor 6 (TRAF6) [47,48]. The expression level of mR-146a varies in different samples (plasma, PBMC, pancreas) and responds to other treatments, ages, and sex [48]. Recently, it was studied that down-regulated miR-146a-5p participates in improving the HG-INS-1 (mouse pancreatic cells in hyperglycemic conditions) cell proliferation and insulin secretion in vitro [49]. The increase in miR-146a expression during inflammation is likely a part of a negative feedback mechanism to prevent excessive production of proinflammatory cytokines [50]. Mir-146a presented a positive correlation with glucose in diabetic patients (correlation = 0.424, *p*-value = 0.002, Figure 5b), but its relationship with inflammatory markers was not detected in our study.

Previous studies reported up-regulated miR-27a expression in the serum of T2DM patients with nephropathy and animal models [51,52]. MiR-27a is recognized as a regulator of adipogenesis and lipogenesis, influencing macrophage polarization by inducing the proinflammatory M1 phenotype, brown adipogenesis, cholesterol homeostasis, and the secretion of inflammatory factors [53,54]. In our study, significant differences between groups were not found. However, we found positive correlations between miR-27a and LPO in diabetic patients and between miR-27a and LPO/AOPP in diabetic patients adjusted for age, gender, and BMI. This possible correlation between the relative expression of miR-27a and markers of oxidative cell damage is in agreement with the findings of Song J. et al., who suggested that miR-27a can contribute to the promotion of oxidative stress by targeting the Nrf2/Keap-1 pathway in the development of the T2DM [53].

Although several studies report a significant increase in miR-21 relative expression in diabetic patients, its relationship with diabetic complications is unclear. Moreover, dynamic miR-21 expression based on the sample type and type of diabetic complication relates to the tissue where it originated. Akpinar et al. reported the decreased expression of miR-21-3p in diabetic patients with increased albuminuria and suggested its association with the development of diabetic nephropathy [55]. Furthermore, it was reported that exosomes derived from macrophages induced by THP-1IL4 (THP1-IL4-exo) polarized primary macrophages to an anti-inflammatory phenotype and control their lipid metabolism. These effects were related to the capacity for THP1-IL4-exo to modulate levels of cellular miRNAs, including an increase in miR-21-5p and miR-146a-5p [56]. MiR-21-5p also improves adipocyte glucose uptake by modulating the PTEN-AKT pathway, protecting against insulin resistance [57]. However, elevated expression of circulating miR-21 has been reported in patients with metabolic syndrome [58] and pre-diabetes [59]. Also, increased miR-21 relative expression was reported in patients with diabetic cardiovascular complications [15], diabetic retinopathy [60], cardiomyopathy [61], in patients with previous major cardiovascular events [25], and with nephropathy [62]. Our results demonstrated that elevated levels of miR-21 were associated with increased LPO and reduced SOD antioxidant activity in diabetic patients, including adjusted for age, gender, and BMI. The positive correlation between miR-21 expression and glycemic parameters (glucose and HbAc1 levels) indicates its participation in diabetes development. Our data also demonstrate that the inclusion of miR-21 in model B1 for predictive risk of diabetes is in accordance with La Sala´s study [63] and confirms the participation of miR-21 as a predictor of oxidative stress damage in patients with a high risk of T2DM.

Finally, the relative expression of miR-484 was significantly increased in patients with T2DM and complications compared to healthy participants in our study population. The seed sequence of the 3ÚTR of mRNA for endothelial nitric oxide synthase (eNOS) is one of the targets of miR-484, and it is reasonable to speculate that miR-484 plays a role in endothelial dysfunction so that it can be involved in cardiovascular disease [64]. Interestingly, miR-484 showed high expression levels in human ischemic heart samples and in diseased endothelial progenitor cells and plasma from patients with coronary atherosclerotic heart disease [65,66]. Dachshund family transcription factor 1 (DACH1) is among the targets of miR-484 [67]. Low DACH1 expression in mouse cardiac endothelial cells promotes worse endothelial cell development and migration, altering vascular endothelial cells’ functioning [68]. Moreover, in rats on myocardial ischemia–reperfusion injury conditions, miR-484 reduces Fis1 protein expression levels [65], a protein that inhibits mitochondria fission. In addition, the involvement of miR-484 in diabetes is known since it may be a regulator of insulin expression—decreasing it in the pancreatic β cells in response to increased glucose [69]. The increase in miR-484 expression is related to MCV risk appearance in our studied population. Moreover, miR-484 is included in the two predictive models for developing cardiovascular complications, considerably increasing its statistical value. Furthermore, among the miRNAs studied in our population, it has the most significant predictive value for developing macrovascular complications. For this reason, further research on specific mechanisms for regulating endothelial injury by miR-484 in T2DM and its complications is required.

A limitation of this study was the reduced sample size per group. However, this study provides valuable information for future studies with more participants. Moreover, longitudinal studies are needed to identify and validate circulating miRNA-based signatures associated with T2DM complications. Additionally, the group of patients with T2DM and vascular complications included a different number of men and women than the other two groups due to randomized recruitment, although there were no significant differences in studied parameters between genders. In the future, clinical departments dedicated to T2DM in hospitals should be linked to basic research areas to send samples and thus make research into molecular biomarkers and their applicability to the patient’s clinical condition more robust. The goal is to make medicine increasingly more personalized. These markers will provide vital information for the search for new treatments.

## 4. Materials and Methods

### 4.1. Study Cohort and Setting

This study employed a cross-sectional design to investigate the complex interplay between miRNAs expressions, oxidative stress markers, inflammatory parameters, and demographic/clinical variables in individuals diagnosed with type 2 diabetes mellitus (T2DM). Participants were recruited from the outpatient clinics of three hospitals in Andalusia, including the Endocrinology and Nutrition Unit of University Hospital San Cecilio of Granada and the Province and the High-Resolution Hospitals of Alcalá la Real and Alcaudete City. A total of 92 participants were divided into 3 groups: (1) 30 controls (CG), (2) 34 patients with T2DM without vascular complications (DG), and (3) 28 patients with T2DM with vascular complications (DG+C), all with their usual treatment. Recruitment was carried out on a discretionary basis, meeting the inclusion and exclusion criteria.

The Ethics Committee of the Andalusian Regional Government, overseeing the two participating hospitals, approved the study protocol. The registration number is 1653-N-21.

All patients provided informed consent. All study groups comprise adult participants over 40 years old and diagnosed with T2DM, according to the American Diabetes Association (ADA) criteria, and at least five years prior, and of both genders.

Control (CG): Healthy volunteers with normal responses to glucose and insulin, without a family history of first-degree diabetes, who did not have any endocrine, vascular, cardiac, or inflammatory pathologies and were not taking any concomitant medication. T2DM patients without vascular complications (DG): Patients with controlled type 2 diabetes mellitus, normal creatinine levels, and no vascular complications. T2DM patients with vascular complications (DG+C): Patients with type 2 diabetes mellitus that had micro- (diabetic retinopathy, nephropathy, and neuropathy) or macrovascular complications (stroke, coronary artery disease, and peripheral arterial insufficiency). All vascular events were documented in the patient’s clinical file or clinical assessment, with your usual treatment assigned by your doctor, which includes antidiabetic drugs.

The general exclusion criteria included the existence of severe liver dysfunction, systemic disorder and evidence of malignant disease, amputations, stage III to V renal insufficiency of the Kdigo obtained by CKD-EPI, or who have other diseases, such as inflammatory, infectious, or autoimmune diseases, or epilepsy, before being diagnosed with T2DM.

To estimate the sample size to be considered, we used the proportion formula: n = [(1.96)2 (p) (1 − p)]/d2, p = First approximation to the population proportion to be estimated in decimals (*p* = 0.153). In this study, the result was 12.4. If we round to 12 and consider that we will describe at least three possible markers, there would be at least 36 patients per group, so we proposed an analysis of 35–36 subjects per group. However, in practice, we could only recruit 34 diabetic patients without complications. In the control group, we recruited 33, but 3 of them were pre-diabetics. The group of T2DM with complications was more challenging to recruit; we recruited 28 participants.

Peripheral blood from the participants was collected from the antecubital veins in tubes containing EDTA after a 10–12 h overnight fast. Following centrifugation at 3500 r/min for 15 min at 4 °C, plasma, and red blood cell aliquots were stored at −80 °C until further testing. Medical history and anthropometric parameters (weight and height) were collected during medical visits.

### 4.2. Biochemical Analysis

Biochemical and hematological parameters were analyzed in the participating hospitals’ clinical analysis service area. Glucose, creatinine, urea, triglycerides, total cholesterol, HDL-cholesterol, and LDL-cholesterol were assayed by colorimetric enzymatic methods using a Cobas c501 analyzer (Roche Diagnostics, Mannheim, Germany). The insulin levels were detected by electrochemiluminescence immunoassay “ECLIA” by a Cobas e801 analyzer (Roche Diagnostics, Mannheim, Germany), and glycosylated hemoglobin (HbA1c) was measured by an ion-exchange high-performance liquid chromatography (IE-HPLC) method using a Tosoh HLC-723-G8 analyzer (Tosoh, Japan). The homeostasis model assessment for insulin resistance (HOMA-IR) was calculated using the equation: [fasting plasma glucose (mg/dL) × fasting insulin (μU/mL)/405.

### 4.3. MicroRNA Expression Analysis

MiRNAs were isolated from 100 µL of plasma using the miRNeasy Serum/Plasma Advanced (Qiagen, Barcelona, Spain, cat. no. 217204), following the manufacturer’s instructions. An external standard, 5 µL of cel-miR-39-30 (assay 478293_mir), was added to evaluate RNA extraction efficiency and as the internal control. Reverse transcription and qPCR for selected miRNAs were performed using the TaqMan MicroRNA Advanced kit (Thermo Fisher Scientific, Waltham, MA, USA) following the manufacturer’s protocol. Mature extracted miRNAs (2 μL) were modified by extending the 3′ end by poly(A) addition; then, the 5′ end was lengthened using an adaptor ligation reaction in a final volume of 15 μL. Finally, the modified miRNAs were reverse transcribed in a final volume of 30 μL, and 5 μL of the RT reaction products were amplified by a miR-Amp reaction, obtaining a uniform pool of cDNA.

Real-time PCR was performed in a final volume of 20 μL, using a mix of TaqMan Fast Advanced Master Mix with miRNA TaqMan Advanced Assays specific for each miRNA (miR-21-5p: assay 477975_mir; miR-126-5p: assay 477888_mir; miR-146a-3p: assay 478714_mir; miR-155-5p: assay 483064_mir; miR-484-5p: assay 478308_mir; miR-27a-3p: assay 478384_mir; miR-210-3p: assay 477970_mir) (Thermo Fisher Scientific, Waltham, MA, USA), through the Agilent Technologies Stratagene Mx3005P System (Agilent Technologies, Madrid, Spain).

All reactions will be performed in triplicate using a QuantStudio 7 Pro Real-Time PCR System, Thermo Fisher Scientific, Waltham, MA, USA, following the manufacturer’s instructions. Data were analyzed using SDS 2.3 and RQ Manager 1.2 software, and relative expression levels of each miRNA were calculated using the 2^−ΔΔCt^ method. A threshold (Cts) less than 33 will be selected for PCR data analysis. The expression of each patient’s miRNA was calculated against the Ct of the control group on the plate.

### 4.4. Determination of Inflammatory Parameters

HCYTA-60K-07 Human Cyto Panel for IL-1β, IL-6, IL-10, IL-8/CXCL8, IL-18, MCP-1/CCL2, and TNF-α (Invitrogen, Madrid, Spain) was used to analyze the profile expression of cytokines in the plasma fraction, following the manufacturer’s instructions. A Luminex 200 system (LX200) of Luminex xMAP technology (Thermo Fisher Scientific, Madrid, Spain) was used to analyze each cytokine based on the corresponding standard curve. The concentrations were determined using Luminex Xponent Solution Software 3.1 (Luminex Corporation, Austin, TX, USA) and were expressed in pg/mL.

### 4.5. Measurement of LPO and AOPP Levels

LPO levels were determined using a commercial colorimetric kit (KB03002, Bioquochem kit, BQC Redox Technologies, Asturias, Spain) that estimates both malondialdehyde (MDA) and 4-hydroxyalkenals. All procedures were conducted according to the manufacturer’s instructions. Absorbance was read at 586 nm, and LPO concentration was expressed in nmol/mL.

AOPP levels were quantified using spectrophotometry on a microplate reader, following the method described by Witko-Sarsat et al. [70]. A standard curve was generated using a chloramine-T solution in the presence of potassium iodide (concentration range: 0–100 nmol/mL) and 20 µL of acetic acid. The absorbance of the reaction mixture was measured at 340 nm against a blank containing 200 µL of PBS, 10 µL of potassium iodide, and 20 µL of acetic acid. AOPP concentration was expressed in nmol/mL of chloramine-T equivalents.

### 4.6. Measurement of GSH and GSSG Levels

Reduced glutathione (GSH) and glutathione disulfide (GSSG) levels were determined in washed red blood cells using O-phthalaldehyde as a fluorescent reagent. Standard curves for GSH and GSSG were utilized for quantification. Fluorescence of the samples was measured at 350 nm excitation and 420 nm emissions using a microplate fluorescence reader (FLx800; BioTek Instruments Inc., Charlotte, VT, USA), following the method described by Hissin and Hilf [71]. The results were expressed as μmol/g Hb.

### 4.7. Measurement of GPx, GRd, SOD, CAT, and G6PD Activities

The glutathione peroxidase (GPx) and glutathione reductase (GRd) activities were spectrophotometrically measured following NADPH oxidation for 3 min at 340 nm in a 96-well plate spectrophotometer (PowerWaveX; BioTek, Washington, DC, USA). The GRd activity was measured using a kit (703202; Cayman chemical, Ann Arbor, MI, USA). Enzyme activities were expressed as μmol/min/g Hb, following the method described by Jaskot et al. [72]. The activity of Cu/Zn-superoxide dismutase (SOD) was measured indirectly by monitoring the absorbance of adrenochrome at 490 nm appearance from adrenaline at a pH of 10.2 [73]. SOD activity was expressed as U/mg Hb (1 unit = 50% inhibition of auto-oxidation of epinephrine). The CAT activity was measured following the decomposition of H_2_O_2_ at 240 nm, according to Aebi´s method [74]. The Erythrocyte G6PD activity was determined by measuring the rate of change in absorbance at 340 nm, due to the reduction in NADP^+^, using the G-6PD kit (Cromakit, S.L., Granada, Spain). All enzyme activities were determined in the erythrocyte fraction.

### 4.8. Nitrite Plus Nitrate Determination

Due to the high instability of nitric oxide, the determination of the nitrites (NOx), the compounds formed after the reaction of nitric oxide with water, must be measured indirectly. Moreover, NOx is rapidly oxidized to nitrates, which should be reduced again to NOx with nitrate reductase to obtain a reliable value of nitric oxide produced during inflammation. The concentration of NOx was measured following the Griess reaction, which converts nitrite into a colored compound, spectrophotometrically detected at 550 nm. Plasma levels of NOx plus nitrates are expressed in mol/L.

### 4.9. Statistics

The data were analyzed using SPSS version 27.0 (WPSS Ltd., Surrey, UK), and graphs were generated using GraphPad Prism v. 6.0 for Windows scientific software (GraphPad Software Inc., La Jolla, CA, USA). The continuous data were tested for normality using the Shapiro–Wilk test, and Levene´s statistics verified the homogeneity of variance. The data were reported as mean ± standard deviation (SD) or mean ± standard error of mean (SEM) for normal and non-normal data, respectively. One-way ANOVA followed by multiple-comparison with post-host test was used for variables with normal distribution. For data that did not fit the normal distribution, the Kruskal–Wallis test was performed. A Pearson´s test was performed to determine correlations between quantitative variables. Receiver operating characteristic (ROC) curves were constructed to evaluate the diagnostic value of miRNAs and other markers. The area under the curve (AUC) and 95% confidence intervals (CI) were calculated to determine specificity and sensitivity. A binomial logistic regression analysis explored the association between plasma miRNAs, oxidative/inflammatory biomarker levels, and the presence of diabetes/complications. Results are displayed as odds ratios (ExpB) and 95% confidence intervals (CIs). Differences were considered statistically significant at *p* values < 0.05.

## 5. Conclusions

To summarize, in the studied Spanish Andalusian population, we found two models with high predictive value for vascular complications presence: (1) HbAc1, creatinine, total cholesterol (TC), lipid peroxidation (LPO), glutathione peroxidase (GPx), superoxide dismutase (SOD), miR-126, miR-484. (2) HbAc1, creatinine, TC, IL-6, LPO, miR-126, miR-484. These results included classical clinical variables and new biomarkers that have reflected the complex pathophysiology of this metabolic disease. Moreover, despite having a limited population, we have described the model associated with macrovascular complications using the binary logistic regression analysis and ROC curve analysis. This model included gender, total cholesterol, GPx, catalase activity (CAT), and miR-484; it exhibited higher predictive values than classical variables. Our findings suggest that further studies should be conducted in a larger population to validate the battery of diagnostic markers for macrovascular complications. Furthermore, in the future, healthcare providers treating diabetic patients should consider using molecular markers to guide their treatment decisions. In this sense, miRNAs could be used as a therapeutic tool.

## Figures and Tables

**Table 1:** Baseline and biochemical parameters of the studied groups.

Parameters	CG (n = 30)	DG (n = 34)	DG+C (n = 28)	*p* Value
**Age**	51.43 ± 1.77	63.47 ± 1.30 ^a^	64.96 ± 1.68 ^b^	^a,b^ *p* < 0.001
**Gender (female/male)**	18/12	18/16	5/23	
**Smoking (yes/no)**	4/26	1/33	4/24	
**Physical activity:**				
**Low intensity**	4	15	17	
**Moderate intensity**	17	19	10	
**High intensity**	9	0	1	
**Weight (kg)**	70.83 ± 12.67	78.81 ± 15.00	86.66 ± 12.18 ^b^	^b^ *p* < 0.001
**BMI (kg/m^2^)**	25.18 ± 3.38	29.84 ± 5.26 ^a^	30.62 ± 4.57 ^b^	^a,b^ *p* < 0.001
**Years of diabetes**	no	13.33 ± 1.85	17.43 ± 2.02	
**HbA1c (%)**	5.37 ± 0.07	6.85 ± 0.16 ^a^	7.11 ± 0.19 ^b^	^a,b^ *p* < 0.001
**Glucose (mg/dL)**	90.33 ± 14.72	123.5 ± 28.24 ^a^	141.9 ± 48.54 ^b^	^a,b^ *p* < 0.001
**Insulin (mU/L)**	6.76 ± 0.74	11.51 ± 1.68 ^a^	11.67 ± 2.02 ^b^	^a,b^ *p* < 0.001
**HOMA-IR Index**	1.50 ± 0.23	3.66 ± 0.63 ^a^	3.99 ± 0.66 ^b^	^a^ *p* < 0.01 ^b^ *p* < 0.001
**Creatinine (mg/dL)**	0.80 ± 0.16	0.84 ± 0.18	0.98 ± 0.35	^b^ *p* = 0.019
**Urea (mg/dL)**	35.41 ± 2.34	42.45 ± 2.47	45.00 ± 4.82	^a^ *p* = 0.035
**Total cholesterol (mg/dL)**	198.8 ± 5.79	168.4 ± 6.30 ^a^	136.3 ± 7.50 ^b,c^	^a^ *p* = 0.003 ^b^ *p* < 0.001 ^c^ *p* = 0.002
**TG (mg/dL)**	90.33 ± 5.73	165.3 ± 23.20 ^a^	140.3 ± 14.29 ^b^	^a^ *p* = 0.01 ^b^ *p* = 0.05
**HDL (mg/dL)**	62.13 ± 2.41	52.65 ± 2.50 ^a^	44.96 ± 2.16 ^b^	^a^ *p* = 0.01 ^b^ *p* < 0.001
**LDL (mg/dL)**	119.5 ± 5.21	85.97 ± 5.72 ^a^	66.63 ± 6.32 ^b^	^a,b^ *p* < 0.001

CG: Control Group. DG: T2DM without complications. DG+C: T2DM with complications. All variables presented a non-normal distribution, and only weight, BMI, and glucose levels had normal distributions. The *p* values were calculated by the Kruskal–Wallis test for non-normally distributed variables. A one-way ANOVA followed by a multiple-comparison post-host test was applied for variables with a normal distribution. Data are presented as means ± standard error of the mean (SEM), and as mean ± standard deviation (SD) for non-normal and normal distributed variables, respectively. ^a^ *p*: DG vs. CG; ^b^ *p*: DG+C vs. CG; ^c^ *p*: DG+C vs. DG.

**Table 2:** Types of medications for patients with Microangiopathies and Cardiovascular Diseases.

Drug Therapy	Microangiopathies (n = 6)	CVD (n = 22)
	21.4%	78.6%
Glucose-lowering medication:		
Metformin	66.7% (4/6)	68.2% (15/22)
Duration of treatment (y)	15.39 ± 2.18	16.23 ± 2.13
Antihyperglycemic agents *	83.3% (5/6)	90.9% (20/22)
Duration of treatment (y)	15.32 ± 2.25	13.00 ± 2.94
Insulin sensitive therapy **	66.7% (4/6)	80.0% (16/22)
Duration of treatment (y)	13.3 ± 2.31	14.66 ± 2.35
Cholesterol-lowering therapy (statins)	66.7% (4/6)	50.0% (11/22)
Duration of treatment (y)	11.57 ± 2.75	17.78 ± 2.31
Anticonvulsants	0.0%	22.7% (5/22)
Duration of treatment (y)		7.92 ± 2.14

* Antihyperglycemic agents include treatment with the second-line glucose-lowering medication (DPP-4 inhibitors, SGLT2 inhibitors, GLP-1 receptor agonists); ** Insulin sensitive therapy includes insulin secretagogues and thiazolidinediones). Painful diabetic neuropathy was treated with anticonvulsants. CVD: Cardiovascular disease. DPP-4: dipeptidyl peptidase-4. SGLT2: sodium-glucose cotransporter 2. GLP-1: glucagon-like peptide type 1, y-years.

**Table 3:** Summary of significant Pearson correlations between oxidative stress and inflammatory markers and biochemical variables for diabetic patients adjusted for gender, age, and BMI.

	Glucose		HbA1c		HOMA-IR		Total Cholesterol		LDL		Creatinine	
	r	p	r	p	r	p	r	p	r	p	r	p
**GPx**	−0.068	0.638	−0.134	0.352	−0.297	0.036	−0.323	0.022	−0.234	0.102	0.066	0.651
**GRd**	0.324	0.022	0.095	0.514	−0.047	0.745	−0.101	0.485	−0.100	0.490	0.087	0.548
**Catalase**	−0.044	0.764	0.020	0.892	0.127	0.258	−0.410	0.003	−0.404	0.004	−0.209	0.146
**G6PD**	0.074	0.608	0.001	0.997	−0.115	0.566	−0.297	0.036	−0.325	0.021	−0.051	0.725
**GSH**	−0.163	0.257	−0.185	0.200	0.030	0.836	−0.302	0.033	−0.288	0.042	0.144	0.319
**IL-8**	0.130	0.424	0.104	0.521	−0.010	0.953	0.330	0.022	0.336	0.061	−0.068	0.677
**IL-10**	−0.167	0.302	−0.177	0.274	−0.365	0.020	0.037	0.819	0.062	0.697	0.062	0.703
**MCP-1**	−0.215	0.183	−0.171	0.290	−0.087	0.595	−0.047	0.771	−0.058	0.714	0.366	0.020

**Table 4:** Summary of significant (*p* < 0.05) Pearson correlations (r) between plasma miRNA levels and oxidative stress measurements in all participants, diabetic patients, and adjusted for age, gender, and BMI.

miRNA	Oxidative Stress Parameters	r-Value	*p*-Value
Diabetic patients			
miR-21	SOD	−0.346	0.009
	LPO	0.266	0.048
miR-126	LPO	0.424	<0.001
miR-27a	LPO	0.321	0.017
miR-210	CAT	0.413	0.001
Diabetic patients were adjusted for age, gender, and BMI.			
miR-21	SOD	−0.289	0.036
miR-126	LPO	0.447	0.001
	AOPP	0.281	0.046
miR-27a	LPO	0.425	0.003
	AOPP	0.288	0.047
miR-210	CAT	0.312	0.029
	LPO	0.292	0.042
miR-484	CAT	0.360	0.039

**Table 5:** Proposed models for predicting the risk of vascular complications in diabetic patients.

Model	AUC (95%)	Exp (B)	ϕ^2^	*p*	R^2^ (Cox y Snell)	R^2^ (Nagelkerke)
Model 1 (Glucose, HbAc1, creatinine, HOMA-IR, total cholesterol, TG, gender)	0.845	0.727	25,724	<0.001	0.363	0.488
Model 2 (HbAc1, creatinine, total cholesterol, LPO, GPx, SOD, miR-126, miR-484)	0.913	0.926	34,093	<0.001	0.481	0.642
Model 3 (HbAc1, creatinine, total cholesterol, IL-6, LPO, miR-126, miR-484)	0.938	0.958	33,863	<0.001	0.513	0.685

**Table 6:** Representation of the predictive values for developing macrovascular diabetic complications among diabetic patients calculated by binary logistic regression analysis and COR curves analysis.

	AUC, (95%)	Exp (B) = OR	*p*
HbAc1	0.488	0.929 (0.530–1.631)	0.797
**Total cholesterol**	0.786	0.968 (0.949–0.987)	<0.001
Creatinine	0.461	0.658 (0.087–4.943)	0.677
HOMA-IR	0.593	1.021 (0.893–1.167)	0.762
LPO	0.491	0.999 (0.339–2.939)	0.998
AOPP	0.524	1.000 (0.981–1.020)	0.990
**CAT**	0.681	1.000 (1.000–1.000)	0.033
**GPx**	0.711	1.051 (1.009–1.094)	0.012
GRd	0.623	1.375 (0.949–1.992)	0.085
GSSG/GSH	0.555	0.138 (0.000–46.142)	0.495
G6DH	0.608	1.192 (0.917–1.550)	0.181
SOD	0.603	1.001 (1.000–1.002)	0.212
NOX	0.556	0.970 (0.921–1.021)	0.194
IL-6	0.529	1.113 (0.860–1.441)	0.416
IL-8	0633	0.750 (0.502–1.121)	0.100
IL-10	0.539	0.973 (0.808–1.171)	0.771
IL-18	0.558	1.006 (0.997–1.015)	0.158
MCP-1	0.485	1.000 (0.995–1.005)	0.968
TNF-a	0.595	0.979 (0.949–1.010)	0.122
miR-21	0.600	1.003 (0.999–1.007)	0.103
miR-126	0.577	0.994 (0.985–1.003)	0.179
miR-146	0.516	1.002 (0.998–1.006)	0.296
miR-155	0.508	1.000 (0.997–1.003)	0.980
**miR-484**	0.674	1.004 (1.001–1.008)	0.008
miR-27a	0.566	1.002 (0.997–1.008)	0.465
miR-210	0.597	1.003 (0.999–1.008)	0.152

## Data Availability

The data supporting reported results are available from the corresponding author (irusanova@ugr.es), on reasonable request and according to privacy and ethical restrictions. Methods and materials described in this manuscript will be freely available to any research to use them for noncommercial purposes.
